# Gastric Ulcers in Alpacas— Clinical, Laboratory, and Pathological Findings

**DOI:** 10.3389/fvets.2022.877257

**Published:** 2022-05-18

**Authors:** Saskia Neubert, Christina Puff, Sven Kleinschmidt, Patricia Kammeyer, Alexandra von Altrock, Michael Wendt, Matthias Gerhard Wagener

**Affiliations:** ^1^Clinic for Swine and Small Ruminants, Forensic Medicine and Ambulatory Service, University of Veterinary Medicine Hannover, Foundation, Hannover, Germany; ^2^Department of Pathology, University of Veterinary Medicine Hannover, Foundation, Hannover, Germany; ^3^Lower Saxony State Office for Consumer Protection and Food Safety, Food and Veterinary Institute Braunschweig/Hannover, Hannover, Germany

**Keywords:** South American camelids, alpaca, gastric ulcer, necropsy, hematology, occult blood

## Abstract

Gastric ulcers are a common finding in post-mortem examinations of South American camelids (SAC), but diagnosis in living animals is often difficult. The aim of this study was to provide an overview of the incidence of gastric ulcers in alpacas, common concomitant diseases, and clinical as well as laboratory findings to facilitate diagnosis for veterinarians. For this purpose, a total of 187 necropsy reports of alpacas were evaluated, including clinical and laboratory findings on the living animal. A total of 23.5% of the animals (*n* = 44) were found to have gastric ulcers, nine were perforated. Compartment 3 was most frequently affected by gastric ulcers. No sex predilection could be detected, but animals 1 year of age and older were more frequently affected by gastric ulcers than animals under 1 year of age. Alpacas with gastric ulcers were presented to the clinic due to different non-specific symptoms. In alpacas with gastric ulcers, significantly more organs or organ systems besides the stomach revealed clinical findings than in animals without gastric ulcers. Of the 44 animals with gastric ulcers, a total of 21 alpacas (47.7%) had a poor nutritional status, but cachexia was not significantly more frequent in animals with gastric ulcers than in other dissected animals without ulcers. Hematologic investigations revealed a significantly lower white blood count and significantly lower segmented neutrophils than in deceased animals without ulcers. Compared to animals discharged after treatment, alpacas that died with gastric ulcers had significantly higher levels of band neutrophils and fewer eosinophils and basophils. Occult blood in feces was found in three of 12 animals with gastric ulcers examined for occult blood. In summary, gastric ulcers are a common problem in SAC, which is difficult to diagnose clinically or by laboratory investigations. As these are often chronic processes involving other organ systems, regular monitoring of the animals' nutritional status and early detection of disease symptoms may help to prevent gastric ulcers.

## Introduction

The keeping of South American camelids (SAC) in Europe is on the rise, and veterinarians thus increasingly have to deal with the treatment of diseases of these exotic species (1–3). The digestive tract is one of the most commonly affected organ systems in SAC (4–7). Especially gastric ulcers regularly occur in SAC (8–10), but they seem to be more common in animals kept outside South America than in the natural habitat of SAC (11). An ulceration is defined on the basis of the affected layers of the gastric wall. Gastric lesions can be histologically classified as ulcers if the necrosis of the epithelium extends to deeper layers than the lamina muscularis mucosae in the case of simple epithelium. Regarding stratified epithelium, it is called an ulcer if the necrosis includes the basement membrane. More superficial lesions are each defined as erosions (12). Gastric ulcers have been described in many animal species, such as pigs (13), horses (14), and ruminants (15–20). Even in wild ruminants, gastric ulcers are regularly observed (21, 22).

The stomach system of SAC with three compartments (C1–C3) has some functional similarities to the four-compartmented stomach of ruminants, but anatomically there are differences (9, 23). Unlike cattle and sheep, there are no papillae in C1 and C2, but all three compartments have glandular areas (10). There is no anatomical separation between the forestomachs and the hydrochloric acid secreting stomach, more precisely the distal 1/5 of C3 (24). Ulcerations are particularly common in C3 but can also be observed in C1 and C2 (8–10). In C3, especially the acid-secreting distal portion of the stomach seems to be affected (6, 9, 25).

Previous studies already evaluated the occurrence of gastric ulcers in llamas and alpacas. In a retrospective analysis of 233 necropsy findings from SAC from Germany, Theuß et al. (4) found a prevalence of 34% for inflammation of the compartments, especially erosive to ulcerative lesions. A perforated gastric ulcer was found in two animals. In a study of 107 necropsy reports from Sweden, gastrointestinal diseases were the most common findings in alpacas, but only four animals had gastric ulcers (3.7%). Both juvenile and adult animals were affected and the lesions were located in C1 and C3 (26). In her thesis, O'Conor Dowd (5) evaluated 359 necropsies of SAC from the Upper Midwest of the USA from 2001 to 2011 and found a prevalence of 6% (*n* = 15/234) for gastric ulcers in C3 in alpacas and a prevalence of 12% (*n* = 15/125) in llamas. Only in some of the cases was the ulceration of C3 the primary cause of death (0.9% in alpacas and 5.6% in llamas). Esophageal and/or C1 ulcers were observed in 2.5% of alpacas and 4.8% of llamas. In contrast, Smith et al. (9) determined the prevalence of C3 ulcers as a cause of death to be about 5% in llamas. In addition, gastric ulcers and erosions were either an incidental observation or a contributing cause of death in more than 20% of the 87 llama cases in Oregon, USA. In a review of necropsy reports from 35 llamas and 58 alpacas in Canada, the authors also concluded that gastric ulcers are a common diagnosis, particularly as an incidental finding, but less frequently as a cause of death from perforation or mycotic colonization in adult animals (27). According to Twomey et al. (28), who investigated 1477 carcass submissions from SAC in England and Wales, gastric ulcers are one of the most frequently diagnosed non-parasitic problems. They were found in 79 of 1477 animals (5.3%). The gastric ulcers were mostly detected in C3; no distinction was made between perforated and non-perforated gastric ulcers. Gastric ulcers seem to be a common diagnosis in adult animals in particular (27), but juvenile animals can also be affected (5, 26). According to Smith et al. (9), the incidence of gastric ulcers in young and adult llamas even seems to be similar, and there seems to be no sex predilection.

It is suspected that stress in particular, through an increase in cortisone, gastric acid, and pepsin as well as a decrease in prostaglandin E production, induce gastric ulcers (8, 9). However, other possible causes, such as the influence of non-steroidal anti-inflammatory drugs (NSAIDs) (29), reflux of duodenal fluid (30), and the influence of the diet, for example forestomach acidosis, which can lead to C1 ulcers (6, 9) as well as reduced feed intake with lack of gastric motility (11) are also discussed. An influence of diet on the development of gastric ulcers could be supported by the fact that llamas and alpacas in South America both consume very few grains and have a low incidence of gastric ulcers (9). The gastric anatomy of SAC could be a predisposing factor for reflux of acidic contents in C3 in the case of an atonic stomach or ileus (24). Gastric ulcers associated with uremia (5, 26, 31) and gastrointestinal tumors (6) have also been described in SAC. The simultaneous presence of debilitating diseases also seems to be a promoting factor for the occurrence of ulcers (4, 8, 9).

If a gastric ulcer is suspected, therapy with proton pump inhibitors can be given to raise the pH value in the C3. In Germany, for food-producing animals such as llamas and alpacas, the active ingredient omeprazole is approved for this purpose. However, oral administration, as used in horses, does not seem to have sufficient effect in SAC (32), and the agent is also insufficiently absorbed when administered rectally (33). Christensen et al. (34) recommend an intravenous administration of 0.4 mg/kg body weight to inhibit C3 acid production for about 6 h. The agent pantoprazole also leads to an increase in the pH value in C3 after subcutaneous or intravenous administration (35). H2-receptor antagonists such as cimetidine and ranitidine, which are used in humans to treat gastric ulcers, are unsuitable for treating gastric ulcers in SAC (29, 34).

However, several authors mention that the clinical symptoms and laboratory findings in animals with gastric ulcers are often non-specific and the diagnosis can frequently only be made post mortem (6, 8, 36). The purpose of this study was a detailed investigation of signs in alpacas with gastric ulcers to help veterinarians diagnose and treat this disease. In contrast to previous studies only dealing with individual aspects of the disease process, this study also included clinical and laboratory data in addition to pathological findings.

## Materials and Methods

### Data Collection

The medical files of alpacas that were presented to the Clinic for Swine, Small Ruminants and Forensic Medicine and Ambulatory Service of the University of Veterinary Medicine Hannover, Germany from January 2005 until the end of November 2021 were evaluated. The patient files of the animals were archived as paper files until August 2016. From then on, all files were archived digitally using “easyVET” (37). Relevant patient data were selected from these files and transferred to an Excel sheet (Microsoft Excel 2016) for evaluation. All data used in this study were collected during veterinary diagnostic procedures after the owners had given written consent.

Data of *n* = 352 alpacas were available for analyses. Of these, 187 animals were necropsied: 136 alpacas (72.7%) were dissected at the Department of Pathology of the University of Veterinary Medicine Hannover, and 51 alpacas (27.3%) were examined at the Food and Veterinary Institute, Lower Saxony State Office for Consumer Protection and Food Safety, Germany. A total of 80 male (42.8%) and 107 female (57.2%) alpacas were examined. The majority of the dissected alpacas were older than 1 year (66.3%, *n* = 124). Most of the animals died during their stay in the clinic (44.9%, *n* = 84), 64 alpacas (34.2%) were euthanized at the clinic, and 39 carcasses (20.9%) were brought to the clinic for diagnostic purposes. The animals had died 1–2 days before presentation.

### Collected Parameters

#### Basic Data on the Animals

For each alpaca, the clinic-ID, the sex (male / female), the age (juvenile: <1 year old/adult: 1 year and older) and, if stated in the medical record, the reasons for the hospitalization were recorded. For each dead animal, it was recorded if it had died spontaneously in the clinic or on the farm or if it had been euthanized.

#### Necropsy

A retrospective review of the necropsy reports was conducted according to the following protocol:

##### Gastric Ulcers

The compartment system was examined for the presence of ulcerations. The alterations were divided into the scores:

0 = no erosions or ulcerations,

1 = erosion(s) (as a preliminary stage of ulceration),

2 = ulceration(s),

3 = perforated gastric ulcer(s).

Furthermore, all animals were divided into “*deceased without gastric ulcer”* [at most preliminary states of ulceration (scores 0 and 1)], and “*deceased with gastric ulcer”* [ulceration or perforated gastric ulcer (scores 2 and 3)]. In addition, for animals with gastric erosions and ulcerations, the compartment or compartments most severely affected were recorded.

##### Nutritional Status

Nutritional status assessed at necropsy was recorded according to the following scheme:

0 = moderate or better,

1 = poor or very poor,

2 = cachexia.

The classification was based on the information recorded in the necropsy reports. In the pathological examination, nutritional status was assessed by the presence of fat depots. In the absence of body and subcutaneous fat depots, nutritional status was rated as poor to very poor. An animal was classified as cachectic if serous atrophy of coronary fat and serous atrophy of bone marrow were also present.

##### Diagnoses

All diagnoses on the respective organs or organ systems were recorded in the Excel sheet using a scoring system. With a score of 0, no findings or findings without clinical relevance were observed in the organ or organ system. Diagnoses of minor or questionable clinical relevance were evaluated as score 1, clinically relevant findings as score 2. In addition, the exact pathological-anatomical diagnosis was noted for scores 1 and 2. Diagnoses interpreted as agonal changes (such as lung congestion) were not recorded. The findings were assigned to the following organs or organ systems: cardiovascular system (heart, vessels); hematopoietic system (bone marrow, lymph nodes, spleen); respiratory system (nasal and sinuses, larynx, trachea, lungs); body cavities (thoracic cavity, abdominal cavity, hernias); liver; genitourinary tract (reproductive organs, urinary organs); musculoskeletal system (bones, joints, muscles, tendons); skin; nervous system (brain and meninges, spinal cord, nerves); eyes; or ears. Since the focus of the study was the gastrointestinal tract, the changes in this organ system were assigned even more precisely to a specific region of the gastrointestinal tract. This involved a classification into mouth (teeth, jaws, oral cavity), esophagus, compartment system and intestine. Body cavity effusions due to cachexia were not recorded. Furthermore, only those lymph node changes were recorded that affected several lymph nodes in different regions of the body (instead of changes in individual lymph nodes due to inflammation in the tributary area). The detection of gastrointestinal parasites during necropsy was also listed using a score of 0 (no detection), 1 (low-grade detection of minor clinical relevance), and 2 (medium to high-grade detection of clinical relevance). In addition, the main diagnoses were recorded as free text for each animal and, if present, general diagnoses [anemia, sepsis, systemic mineralization, systemic mycosis, cachexia and uremia (diagnosed by examination of the aqueous humor for urea concentration)] were noted as free text.

#### Hematologic Parameters

From all living animals presented to the clinic, blood samples were taken from the jugular vein as part of the general clinical examination (EDTA Monovette 9 mL K3E, Sarstedt AG & Co. KG, Nümbrecht, Germany). Blood samples were either processed directly or stored at 4°C when the animals were presented at night or during the weekend for up to 3 days. This initial blood sample was recorded from all dissected alpacas as far as it was available. In addition, as a control group, the values of the initial blood sample from 165 alpacas that were later discharged as cured were noted. The following blood values were recorded: packed cell volume (PCV) [l/l], hemoglobin (Hb) [g/l], white blood count (WBC) [G/l], lymphocytes [G/l], segmented neutrophils [G/l], band neutrophils [G/l], eosinophils [G/l], basophils [G/l], monocytes [G/l]. The blood values were determined as described previously (38).

#### Fecal Samples

For animals that were dissected, it was additionally noted whether a fecal sample was taken for quantification of gastrointestinal nematodes (GIN) and/or for testing for occult blood during the initial clinical examination. Existing results were included in the Excel sheet. The methods used for quantifying of GIN and testing for presence of occult blood have been described previously (39). Detection of 1–50 GIN eggs per gram of feces was classified as low-grade infestation, 51–100 GIN eggs/g feces as medium-grade infestation, 101-500 GIN eggs/g feces as high-grade infestation, and over 500 GIN eggs/g feces as very severe infestation.

### Statistical Analyses

Data analyses were carried out using Microsoft Excel 2016 and R Statistics version 3.6.1. Chi-squared tests with Yates' Correction were used to investigate a potential relationship between sex (male and female) and the occurrence of gastric ulcers, and between age (juvenile and adult) and the occurrence of gastric ulcers. A series of Chi-squared tests was conducted to compare the blood values of deceased alpacas with gastric ulcers with blood values of a norm population based on age- and sex-specific reference intervals. In the norm population, the reference range was given as the 10th and 90th quantiles; therefore, ten percent of the reference population was assumed to fall both below and above the norm reference interval. Multiple binomial regressions and multiple linear regressions were used to test whether dead animals with gastric ulcers were more likely to show cachexia than dead animals without gastric ulcers, whether they had more clinically relevant affected organ systems (score 2), which systems were more likely to be affected, and whether they had significantly different blood values than both dead animals without gastric ulcers or discharged animals. For analyses comparing dead animals with and without gastric ulcers, *age* (see Gastric Ulcers at Necropsy) and *gastric ulcer* were entered as binary independent variables. For blood value analyses comparing dead animals with gastric ulcers with dead animals without gastric ulcers and with discharged animals, *age* was entered as a binary independent variable (see Gastric Ulcers at Necropsy) and *deceased without gastric ulcer vs. deceased with gastric ulcer*, as well as *discharged vs. deceased with gastric ulcer* were entered as dummy variables. For numeric variables, outliers that were more than three standard deviations above the mean were replaced by the mean value plus three standard deviations.

## Results

### Gastric Ulcers at Necropsy

**Figure 1:**
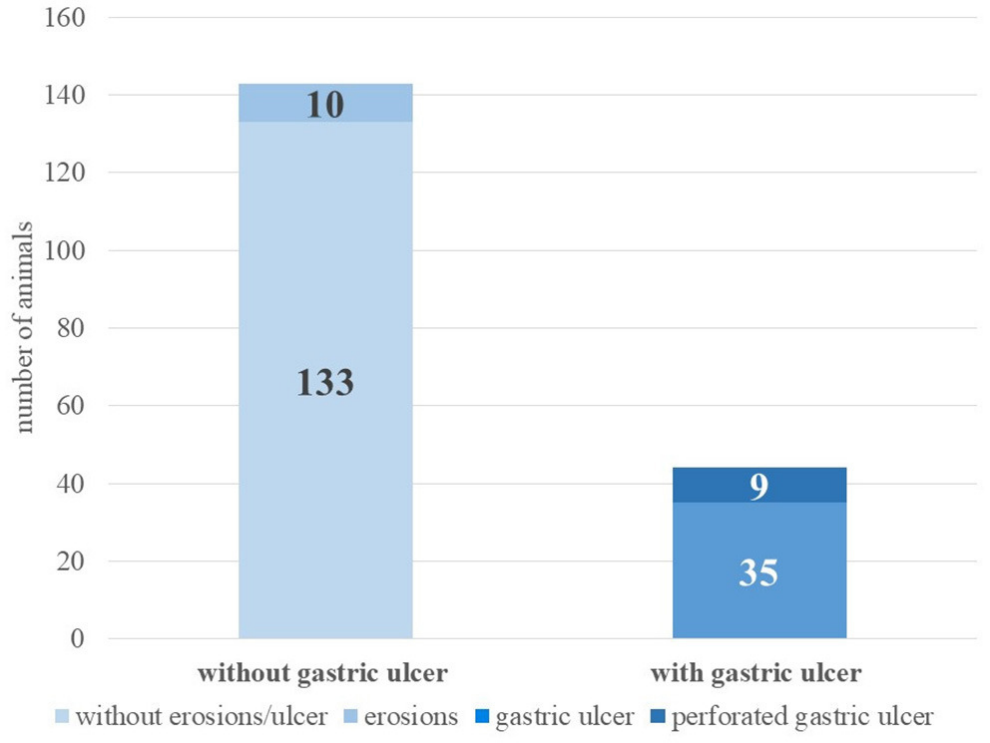
Incidence of gastric ulcers at necropsy (alpacas, *n* = 187).

Ulcerative changes in the compartments were detected in 23.5% of the 187 animals (*n* = 44), nine of them perforated. Erosions were found in another 10 animals (see Figure 1).

Of the 107 female and 80 male alpacas, 22 animals of each sex had gastric ulcers. Deceased females were not significantly more or less likely to have gastric ulcers than deceased males [$\chi {{}^{\text{2}}}_{\left(1\right)}$ = 0.87, *p* = 0.35]. However, adult animals were significantly more likely to have gastric ulcers than juvenile animals [$\chi {{}^{\text{2}}}_{\left(1\right)}$ = 7.14, *p* < 0.01]. The odds for adults having gastric ulcers were 3.38 [1.36, 9.62] times higher than those for juveniles (see Table 1). Therefore, age was included as a binary control variable in all regression analyses.

**Table 1:** Incidence of gastric ulcers in juvenile and adult alpacas (*n* = 187).

**Age group**	**Gastric ulcers**	
	**No**	**Yes**
Juvenile	56 (89%) (male: 24; female: 32)	7 (11%) (male: 3; female: 4)
Adult	87 (70%) (male: 34; female: 53)	37 (30%) (male: 19; female: 18)
Total	143	44

*χ^2^(1) = 7.14, p < 0.01*.

The third compartment (C3) was most commonly affected by ulceration (61.4%, *n* = 27). In seven animals (15.9%), the ulcerative lesions were mainly in C1, and in four animals (9.1%) in C2. In six animals (13.6%), similar ulcerative lesions were found in several compartments (C1/C2: three animals; C1/C3: two animals; C1/C2/C3: one animal). A perforated gastric ulcer was observed in nine alpacas; these were found in eight animals in C3 and in one animal in C2. All nine animals with perforated gastric ulcers had consecutive peritonitis. In 10 animals, only erosions were detected, which were located mainly in the third compartment (*n* = 8) and rarely in C1 (*n* = 2).

The nutritional status was less than moderate (score > 0) in 21 alpacas with gastric ulcers (47.7%). A total of 60 alpacas (32.1%) were diagnosed with cachexia, 13 of these animals had gastric ulcers. A multiple binomial regression showed that deceased animals with gastric ulcers did not show cachexia (score 2) statistically significantly more often than deceased animals without gastric ulcers (see Table 2).

**Table 2:** Binomial regression estimation for cachexia (*n* = 187).

**Variable**	***B* (*SE*)**	***OR* [95% CI]**
(Constant)	−0.89** (0.29)	
Adult	0.29 (0.34)	1.33 [0.69, 2.66]
Deceased with gastric ulcers	−0.22 (0.38)	0.80 [0.37, 1.67]

*B, Regression coefficient; SE, Standard error; OR, Odds ratio; CI, Confidence interval*.

R^2^ = 0.01 (Nagelkerke). p < 0.10, *p < 0.05, **p < 0.01, ***p < 0.001.

In addition to cachexia as a general diagnosis, anemia was diagnosed due to the macroscopic appearance during dissection in five animals with gastric ulcers (one of them in combination with high-grade endoparasitosis). Septicemic processes were suspected in seven animals. In another four animals, ulcerations were associated with systemic mineralization and vitamin D hypervitaminosis (three of them additionally with uremia). In two animals, uremia was present without systemic mineralization but with renal alterations.

In six cases, gastric ulcers were tumor-associated (squamous cell carcinoma: *n* = 3 in C1; adenocarcinoma: *n* = 3 in C3). Fungal hyphae were detected in the ulcerations of four animals.

Of four alpacas with changes only in the compartments but not in other organs, three were additionally cachectic and one was found to have forestomach acidosis [evaluated by measuring the pH of the compartments using pH test strips (pH-Fix 0-14 PT, Macherey-Nagel GmbH & Co. KG, Düren, Germany)]. However, in almost all alpacas with gastric ulcers (90.9%, *n* = 40), clinically relevant pathological changes were also found in other organs or organ systems.

A multiple linear regression analysis showed that significantly more organs or organ systems beyond the compartments had pathological findings in deceased animals with gastric ulcers as opposed to in deceased animals without gastric ulcers (see Table 3).

**Table 3:** Multiple linear regression estimation for number of clinically relevant affected organs/organ systems.

**Variable**	***B***	***SE***	**β**
(Constant)	1.42***	0.16	
Adult	0.02	0.21	0.01
Deceased with gastric ulcers	0.47*	0.23	0.36
*n*	187		
*R^2^*	0.02		

*B, Regression coefficient; SE, Standard error; β, standardized coefficient*.

^†^p <0.10, *p < 0.05, **p < 0.01, ***p < 0.001.

Overall, alpacas that died with gastric ulcers had pathological findings of the hematopoietic system and body cavities significantly more often than deceased animals without gastric ulcers. Furthermore, the former had pathological findings of the respiratory tract and esophagus marginally significantly more often than deceased animals without gastric ulcers (see Table 4).

**Table 4:** Multiple binomial regression estimation for clinically relevant affected organs/organ systems (*n* = 187).

**Variable**	**Cardiovascular system**		**Hematopoietic system**		**Respiratory system**		**Body cavities**		**Liver**		**Genitourinary tract**	
	***B* (*SE*)**	***OR* [95% CI]**	***B* (*SE*)**	***OR* [95% CI]**	***B* (*SE*)**	***OR* [95% CI]**	***B* (*SE*)**	***OR* [95% CI]**	***B* (*SE*)**	***OR* [95% CI]**	***B* (*SE*)**	***OR* [95% CI]**
(Constant)	−1.91*** (0.38)		−4.53*** (1.04)		−1.00*** (0.29)		−1.62*** (0.33)		−1.76*** (0.36)		−1.96*** (0.38)	
Adult	−0.28 (0.50)	0.76 [0.29, 2.08]	1.60 (1.07)	4.95 [0.89, 92.54]	−0.42 (0.37)	0.66 [0.32, 1.36]	0.10 (0.40)	1.11 [0.51, 2.49]	1.00* (0.42)	2.72 [1.24, 6.48]	0.04 (0.47)	1.04 [0.42, 2.73]
Deceased with gastric ulcers	−0.16 (0.60)	0.85 [0.23, 2.56]	1.74** (0.60)	5.68 [1.81, 19.81]	0.69^†^ (0.39)	1.99 [0.92, 4.27]	1.16** (0.39)	3.19 [1.49, 6.85]	−0.34 (0.42)	0.71 [0.30, 1.57]	0.26 (0.50)	1.30 [0.47, 3.34]
*R^2^*	0.01		0.19		0.03		0.08		0.05		0.00	
**Variable**	**Musculoskeletal system**		**Skin**		**Nervous system**		**Mouth**		**Esophagus**		**Intestine**	
	***B*** **(*****SE*****)**	***OR*** **[95% CI]**	***B*** **(*****SE*****)**	***OR*** **[95% CI]**	***B*** **(*****SE*****)**	***OR*** **[95% CI]**	***B*** **(*****SE*****)**	***OR*** **[95% CI]**	***B*** **(*****SE*****)**	***OR*** **[95% CI]**	***B*** **(*****SE*****)**	***OR*** **[95% CI]**
(Constant)	−3.01*** (0.60)		−4.05*** (1.01)		−1.14*** (0.31)		−2.25*** (0.44)		−4.41*** (1.04)		−1.48*** (0.33)	
Adult	0.29 (0.71)	1.34 [0.36, 6.42]	1.46 (1.09)	4.29 [0.73, 81,71]	−1.39** (0.50)	0.25 [0.09, 0.65]	−0.42 (0.58)	0.66 [0.21, 2.13]	0.81 (1.12)	2.26 [0.34, 44.28]	−0.20 (0.41)	0.82 [0.37, 1.87]
Deceased with gastric ulcers	0.15 (0.72)	1.16 [0.24, 4.37]	−1.03 (1.09)	0.36 [0.02, 2.12]	−1.61 (1.05)	0.20 [0.01, 1.05]	−0.03 (0.69)	0.97 [0.21, 3.46]	1.39^†^ (0.80)	4.01 [0.83, 21.76]	0.29 (0.45)	1.34 [0.54, 3.15]
*R^2^*	0.00		0.01		0.15		0.01		0.09		0.01	

*B, Regression coefficient; SE, Standard error; OR, Odds ratio; CI, Confidence interval*.

^†^p < 0.10, *p < 0.05, **p < 0.01, ***p < 0.001.

### Clinical Presentation of Animals With Gastric Ulcers

In the 44 alpacas that were presented to the clinic and that revealed gastric ulcers at necropsy, general weakness or recumbency (in 68% of the animals, *n* = 30) and anorexia (43%, *n* = 19) were the most common symptoms observed by the animal owners. Colic was noted in about a quarter of the animals (27%, *n* = 12). Less frequently, the alpacas showed diarrhea and emaciation (18% each, *n* = 8). Other sporadically mentioned reasons for presentation were anemia (*n* = 3), pyrexia (*n* = 2), a distended abdomen (*n* = 2) or melena (*n* = 1). In 12 animals, the owners described other symptoms not associated with gastric ulcers. One animal had no symptomatic disorders and died peracutely.

### Gastric Ulcers and Hematology

The average blood values of deceased alpacas with gastric ulcers and their comparison with blood values of a norm population (40) are given in Table 5. PCV, hemoglobin, WBC, lymphocytes, segmented neutrophils, eosinophils, and monocytes of deceased animals with gastric ulcers were significantly more often below the respective reference range than in animals of a norm population. About 40–50% of the animals with gastric ulcers revealed deviations in PCV, hemoglobin, WBC, lymphocytes, segmented neutrophils, and monocytes below the reference values corresponding to age and sex (for the age- and sex-specific reference ranges, see Supplementary Material 1). Eosinophils were below the reference interval in 94% of the animals with gastric ulcers. The band neutrophils were above the reference ranges in about 94% of the animals, and were significantly more often above the reference ranges than in alpacas of a norm population. About 20–30% of the deceased animals with gastric ulcers had WBC and segmented neutrophils above the age- and sex-specific reference range.

**Table 5:** Average blood values of deceased alpacas with gastric ulcers (*n* = 44) and comparison with a norm population.

**Parameter**	***n***	**Mean (SD)**	**Comparison with a norm population**						
			**Normal reference interval^**a**^**	**Animals below^**b**^ (%)**	***X^2^*(1)**	***OR* [CI]**	**Animals above^**b**^ (%)**	***X^2^*(1)**	***OR* [CI]**
PCV [l/l]	36	0.25 (0.10)	0.26–0.37	50.00	26.27***	8.6 [3.36, 22.89]	11.11	0.00	1.10 [0.24, 3.88]
Hemoglobin [g/l]	36	114.83 (44.62)	110–166	47.22	23.15***	7.71 [3.00, 20.49]	8.33	0.00	0.80 [0.14, 3.15]
WBC [G/l]	35	13.64 (15.77)	7.3–16	40.00	15.42***	5.76 [2.18, 15.53]	22.86	2.84^†^	2.58 [0.84, 7.54]
Lymphocytes [G/l]	35	1.94 (1.85)	1.1–5.9	48.57	24.24***	8.13 [3.14, 21.79]	5.71	0.24	0.53 [0.06, 2.54]
Segmented neutrophils [G/l]	35	7.93 (10.03)	2.9–9.3	42.86	18.20***	6.47 [2.47, 17.38]	28.57	6.14*	3.47 [1.22, 9.74]
Band neutrophils [G/l]	35	2.67 (5.78)	0–0.2				94.29	91.24***	136.44 [29.67, 1313.26]
Eosinophils [G/l]	35	0.06 (0.10)	0.1–3.6	94.29	91.24***	136.44 [29.67, 1313.26]	0.00	2.63	0.00 [0.00, 1.14]
Basophils [G/l]	35	0.03 (0.04)	0–0.3				0.00	2.63	0.00 [0.00, 1.14]
Monocytes [G/l]	35	0.60 (1.19)	0.1–0.9	40.00	15.42***	5.76 [2.18, 15.53]	17.14	0.69	1.81 [0.52, 5.65]

*PCV, Packed cell volume; WBC, White blood count; SD, Standard deviation; OR, Odds ratio; CI, Confidence interval*.

*^a^Based on published data (40); combined range of three reference intervals (juvenile, male, female)*.

*^b^Based on the age- and sex-specific reference interval; for the specific reference ranges, see Supplementary Material 1*.

p < 0.10, *p < 0.05, **p < 0.01, ***p < 0.001.

Multiple linear regressions were carried out to compare deceased animals with gastric ulcers with those deceased without gastric ulcers and discharged animals. Deceased animals with gastric ulcers had a significantly lower WBC than deceased animals without gastric ulcers and had significantly lower segmented neutrophils. Compared to animals discharged alive, animals that died with ulcers had significantly higher values of band neutrophils and lower values of eosinophils and basophils. This significant distinction was not apparent between deceased alpacas with gastric ulcers and deceased alpacas without gastric ulcers (see Table 6). For numerical comparison of average blood values of deceased animals with gastric ulcers and deceased animals without gastric ulcers and discharged animals, see Supplementary Material 2.

**Table 6:** Multiple linear regression estimation for blood values.

**Variable**	**Packed cell volume [l/l]**			**Hemoglobin [g/l]**			**White blood count [G/l]**			**Lymphocytes [G/l]**			**Segmented neutrophils [G/l]**		
	***B***	***SE***	**β**	***B***	***SE***	**β**	***B***	***SE***	**β**	***B***	***SE***	**β**	***B***	***SE***	**β**
(Constant)	0.29***	0.02		133.46***	7.38		10.48***	1.66		2.56***	0.29		5.65***	1.36	
Adult	−0.04***	0.01	−0.52	−20.95***	4.92	−0.57	1.62	1.12	0.20	−0.83***	0.19	−0.59	1.92*	0.92	0.29
Discharged alive vs. deceased with gastric ulcers	−0.00	0.01	−0.01	−3.12	6.57	−0.09	0.35	1.47	0.04	0.37	0.25	0.26	0.78	1.20	0.12
Deceased without gastric ulcers vs. deceased with gastric ulcers	−0.01	0.02	−0.10	−6.17	7.06	−0.17	3.34*	1.58	0.42	−0.03	0.27	−0.02	3.41**	1.29	0.52
*n*	306			305			292			290			290		
*R^2^*	0.05			0.06			0.03			0.07			0.04		
**Variable**	**Band neutrophils [G/l]**			**Eosinophils [G/l]**			**Basophils [G/l]**			**Monocytes [G/l]**		
	***B***	***SE***	***β***	***B***	***SE***	***β***	***B***	***SE***	***β***	***B***	***SE***	***β***
(Constant)	1.71***	0.30		−0.07	0.12		0.00	0.02		0.47***	0.10	
Adult	0.18	0.20	0.13	0.15^†^	0.08	0.26	0.02	0.01	0.23	0.00	0.07	0.01
Discharged alive vs. deceased with gastric ulcers	−1.05***	0.26	−0.73	0.50***	0.10	0.86	0.05**	0.02	0.51	−0.14	0.09	−0.30
Deceased without gastric ulcers vs. deceased with gastric ulcers	−0.38	0.28	−0.26	0.16	0.11	0.27	0.01	0.02	0.15	−0.08	0.09	−0.18
*n*	290			290			290			290		
*R^2^*	0.08			0.14			0.06			0.01		

*B, Regression coefficient; SE, Standard error; β, standardized coefficient*.

^†^p < 0.10, *p < 0.05, **p < 0.01, ***p < 0.00.

### Gastric Ulcers and Fecal Occult Blood

In 35 animals, fecal samples were analyzed for occult blood; results are shown in Table 7. Ten of 44 animals with gastric ulcers were found to have acute mucosal hemorrhages at necropsy, four of which were tested for occult blood during hospitalization. Two were found to be positive and two negative. In the three alpacas with gastric ulcers and where occult blood was detected, only few gastrointestinal nematode eggs were detected in the feces (low-grade worm infestation in each case). Two of these alpacas had perforated gastric ulcers. Of the 10 alpacas tested positive for occult blood but without any ulcers, eight animals had a medium-grade (*n* = 2), high-grade (*n* = 4), or very severe (*n* = 2) worm infestation. In two of the animals, no or few GIN eggs were detected in the feces. In nearly all 13 animals without gastric ulcers and without any detected occult blood, no or few GIN eggs were detected; one animal had very severe worm infestation.

**Table 7:** Occurrence of occult blood in the feces (*n* = 35).

**Gastric ulcers**	**Occurrence of occult blood**	
	**No**	**Yes**
No	13	10
Yes	9	3

## Discussion

Compared to previous studies (4, 8, 9, 27), the results of our study showed a rather high proportion of deceased alpacas with gastric ulcers (23.5%). When including animals with erosions, the proportion was even higher (28.9%). According to the pathological definition (12), a lesion is only called an ulceration when certain layers of the gastric wall are affected, but in cows, a classification scheme has been established in which even erosions with minimal mucosal defects are considered as gastric ulcers (17, 41). Since there is no corresponding classification system for SAC, Hund and Wittek (8) recommended adopting the existing scheme used for cows for llamas and alpacas. In the present study, however, the necropsy reports were evaluated retrospectively, the affected layers of the gastric wall and their appearance not being reported in detail. Therefore, the assessment was made with a simplified score system with a classification into no lesions, erosion, ulceration, and perforated ulceration. Since the literature often refers only to gastric ulcers and it is unclear whether erosions are also included, the focus of this study was on animals with ulcerations as defined by Klopfleisch and Gruber (12), which means mucous membrane alterations extend to deeper layers than the lamina muscularis mucosae.

No sex predisposition for the occurrence of gastric ulcers was found in the investigated animals, which is in line with the results of Smith et al. (9). Furthermore, according to the investigations of Smith et al. (9), gastric ulcers occur equally frequently in juvenile and adult animals, which could not be confirmed in our study. However, Shapiro et al. described that gastric ulcers are a frequent finding, especially in adult animals (27). The frequent occurrence of gastric ulcers, especially in C3 rather than in C1 and C2 in the present study is also consistent with existing research data (5, 8, 10, 28). Based on the necropsy reports, however, it could not be determined whether the acid-secreting distal portion of C3 was particularly affected, as described in some former studies (6, 9, 25).

An assumption about the etiology of the gastric ulcers in the examined animals cannot be made on the basis of the retrospectively evaluated data, as uniform preliminary reports as well as standardized data were not available. In a survey of 255 owners of SAC in Germany, almost half of the farms used their animals for trekking tours and a quarter of them used them for therapeutic purposes (3). The extent to which activities, involving handling, contact with strangers, and possibly transport, lead to stress reactions in some animals can only be speculated. Housing conditions can also cause stress in the animals, for example, in the case of ranking fights in male groups or a lack of possibilities to avoid confrontations for lower-ranking animals (42). Stress as a possible cause of gastric ulcers in SAC is suspected in other studies (8, 9, 43) and may also have played a role in the presented cases. Further research on gastric ulcers in SAC with comprehensive anamneses would be desirable.

In some examined carcasses, uremic gastritis due to renal changes may be considered as a cause of the ulcers, as has been suspected in other studies (5, 26). In this context, vitamin D intoxication must be considered as a cause of systemic calcification, especially nephrocalcinosis, leading to uremia (44, 45). Such an oversupply of vitamin D was found in four animals, which already featured in detailed case reports (31, 46). Other authors already reported on tumor-associated gastric ulcers. According to Cebra (6), gastrointestinal tumors in SAC can lead to focal ulcerations in the entire gastrointestinal tract, with squamous cell carcinomas occurring most frequently, usually in the first compartment. This was confirmed in different case reports and in the animals in the present study, but other localizations of the tumor are also possible (47, 48). However, adenocarcinomas were reported in the present study with the same frequency as squamous cell carcinomas, with all adenocarcinomas located in the C3. A study from California found oral, esophageal, and gastric ulcers to be associated with the presence of *Fusobacterium necrophorum*, but it could not be determined whether the pathogen led to the mucosal lesions as the primary agent or whether it colonized in already damaged tissue as a secondary agent (49). In the present study, microbiological examinations of gastric ulcers were not included, so further research on this topic is needed.

It is remarkable that gastric ulcers rarely occurred as a sole finding. Overall, other organs and organ systems were even statistically significantly more frequently affected in animals with gastric ulcers than in deceased alpacas without ulcers. Correspondingly, other authors also found gastric ulcers rarely occurring as sole findings; they therefore often described them as incidental findings unless they were severe and led to peritonitis and sepsis (9, 27). Likewise, in the evaluation of necropsy findings by Theuß et al. (4), many animals with gastric ulcers suffered from another underlying disease. This statement is supported by the fact that many animals were cachectic, which indicates a chronic disease process. However, it is often not possible to determine whether the cachexia was caused by the gastric ulcer or whether the gastric ulcer is a consequence of the cachexia. In addition, pathological changes in other organs or an insufficient diet can lead to cachexia. In our study, many alpacas had a poor to cachectic nutritional condition, which did not indicate gastric ulcers. In order to detect emaciation as a possible sign for disease processes in time, a regular recording of the Body Condition Score (BCS) may help (38, 50, 51).

The fact that body cavities and the hematopoietic system were significantly more frequently affected in alpacas with gastric ulcers was expected; all animals with perforated ulcers revealed peritonitis, some of which was accompanied by sepsis. In addition, the gastrointestinal tumors were associated with gastric ulcers, and since some of the gastrointestinal tumors metastasized, many lymph node-associated changes were thus seen in animals with gastric ulcers. Hughes and Mueller (52) and Kramer et al. (53) reported that gastric ulcers can be a portal of entry for various fungal spores and can thus lead to mycotic pneumonia, sometimes also to systemic mycoses. Although fungal spores were detected in the ulcerations of four animals, no systemic mycoses or mycotic pneumonias were found in animals with gastric ulcers.

Since gastric ulcers obviously have a multifactorial pathogenesis, it must rather be assumed that a debilitation of the whole organism can have a predisposing effect on gastritis and on increased alterations in various organs. It remains unclear when in the course of the disease the gastric ulcer occurred, whether as a consequence or as a cause of other findings.

Just as the animals with gastric ulcers were usually found to have several concurrent diseases at necropsy, the clinical picture of the animals was also usually non-specific. Symptoms like general weakness and recumbency as well as anorexia occurred, which can be present in many diseases. These non-specific symptoms were also reported by other authors. Smith et al. found colic and recumbency in one third of the animals, depression and anorexia were even more frequent (9). According to Zanolari et al. (11), many gastric ulcers even occur without clinical symptoms. Smith et al. (41), who studied abomasal ulcers in cows, noted symptoms with varying frequency depending on the type of ulcer. According to them, for example, melena and pale mucous membranes indicating anemia are found mainly in cows in which the gastric ulcers involve a large vessel and bleed profusely. Since the C3 is well-vascularized in SAC (54), it seems very likely that bleeding ulcers will occur. According to Fowler (10), melena and anemia are not observed, as bleeding gastric ulcers, as they occur in ruminants, do not occur in llamas and alpacas. Smith et al. (9) and Theuß et al. (4) did not detect melena in any of the examined animals. In contrast, Cebra (6) described melena in camelids, but as a very rare symptom, more often associated with a tumor. Consistent with this former study, anemia and melena were hardly ever observed by the owners in the present study, and acute bleeding from the gastric mucosa was only observed in 10 animals.

When presenting animals with non-specific gastrointestinal symptoms, differential diagnoses such as uterine torsion, urolithiasis, constipation, plant intoxication, neoplasia, and others should also be taken into account (9).

As further diagnostics, only indirect procedures, such as a test for occult blood or a hematologic examination are discussed. A gastroscopy is anatomically rarely possible in SAC (8, 9).

According to Cebra (6), hematologic changes in animals with gastric ulcers are non-specific, anemia may occur, but it may also indicate other diseases. Although most blood values of alpacas with gastric ulcers are on average within the reference ranges, a large scattering is noticeable and many blood values are significantly more often below (PCV, hemoglobin, WBC, lymphocytes, segmented neutrophils, eosinophils, monocytes) and significantly more often above (band neutrophils) the age- and sex-specific reference ranges in deceased alpacas with gastric ulcers than in alpacas of a norm population. The fact that the PCV was below the reference range in 50% of the animals with gastric ulcers, but not significantly different from deceased animals without gastric ulcers or discharged animals, was therefore not surprising, also due to the fact that acute bleeding is rarely observed. Although an increased WBC could be expected in inflammatory processes, this was not reflected in the blood values of the animals with gastric ulcers. Compared to deceased animals without gastric ulcers, they even had a significantly lower WBC and a significantly lower proportion of segmented neutrophils. Furthermore, 40% of the animals with gastric ulcers had a WBC below the normal alpaca reference range. Serous atrophy of the bone marrow due to emaciation may lead to leukopenia (55). Another possible explanation for this might be that increased consumption of leucocytes exceeds the compensatory capacity of the bone marrow in these animals (56). A similar picture is also seen in cows with perforated gastric ulcers (20). Likewise, Smith et al. (9) describe that although an increase in WBC is usually seen initially in animals with perforation of gastric ulcers, this develops over time into leukopenia with a left shift. This is consistent with the increased levels of band neutrophils, i.e., immature neutrophils found in animals with gastric ulcers. These were significantly higher than those found in animals discharged alive, and were above the normal alpaca reference interval in 94% of the animals with gastric ulcers. The fact that this significance was not seen between deceased animals with gastric ulcers and deceased animals without gastric ulcers is probably due to the fact that these animals suffered from other wasting diseases, which led to shifts in the leucocyte fractions. It remains unclear why deceased animals with gastric ulcers had significantly lower proportions of eosinophils and basophils compared to those animals discharged alive, and why nearly all animals with gastric ulcers (94.92%) had proportions of eosinophils below the normal alpaca reference interval. The lower proportion could be due to stress (56). However, since eosinophils and basophils are generally present in low numbers in a blood smear, a single over- or under-recognized cell during manual differentiation may have led to an error. Furthermore, it should be noted that other authors reported other reference ranges for eosinophils, according to which many of the animals with gastric ulcers would be within the reference range (57, 58).

When interpreting the hematologic results, it should be taken into account that in most animals with gastric ulcers, other organs were also affected, which could have falsified the interpretation. In addition, the blood samples of the animals that were discharged alive were taken on the first day of hospitalization, so there were undoubtedly animals that had diseases and thus showed an altered blood count. These animals may also have had gastric ulcers that went undetected in the living animals. Therefore, the results need to be interpreted with caution. Another source of uncertainty are the hematologic reference intervals for alpacas. Various studies on hematologic values of alpacas have been published (40, 57–59), but they are limited by low numbers of reference individuals and different methods used. It should further be noted that in the reference intervals of Hengrave Burri et al. (40), alpacas under 6 months of age were classified as juveniles. In the present study, all animals under 1 year of age were classified as juvenile; two were over 6 months of age and were nevertheless compared with the reference ranges for juvenile alpacas.

In addition to hematologic investigations, a fecal occult blood test can help diagnose bleeding gastric ulcers. However, like the rare detection of melena, occult blood is also rarely found in the feces of SAC with gastric ulcers (9, 10). In this retrospective study, unfortunately only a small proportion of animals were tested for occult blood, and only a few animals with gastric ulcers had a positive result. Even in four alpacas with evidence of acute bleeding in the compartments, the results of the test were positive in only two cases. Cebra (6) and Bauerstatter et al. (60) also reported doubtful results when testing llamas and alpacas for occult blood.

As a differential diagnosis to blood loss due to gastric ulcers, an infection with gastrointestinal strongylids, especially *Haemonchus contortus*, which occurs with a high prevalence in the SAC population, must always be considered (61). Due to irritation of the gastric mucosa, blood loss into the gastric lumen may occur. However, in the animals with gastric ulcers and evidence of occult blood, endoparasitosis was excluded.

The results show that testing SAC for occult blood is of limited use as a diagnostic tool, and a negative finding does not rule out the presence of a gastric ulcer. Nevertheless, further research with a larger number of animals is needed.

Another possible diagnostic method for gastric ulcers is the examination of forestomach fluid for bile acid content. Nonetheless, this method seems to have little significance in relation to gastric ulcers (30) and was not performed in this study. Diagnostic laparotomy should only be considered as a last measure (9).

## Conclusion

In summary, the diagnosis of “gastric ulcer” in living SAC is very difficult. Symptoms are often non-specific and diagnostic tools are limited. The occult blood test rarely leads to positive results in animals with gastric ulcers, as ulcerations with severe blood losses hardly ever occur. Hematologic investigations also do not provide clear evidence of the presence of gastric ulcers. The pathological findings in alpacas with gastric ulcers indicate a multifactorial pathogenesis and frequently a chronic process.

To prevent the occurrence of gastric ulcers, stress in particular should be avoided in the animals. In addition, the SAC should be monitored regularly in order to detect disease symptoms at an early stage.

## Data Availability Statement

The raw data supporting the conclusions of this article will be made available by the authors, without undue reservation.

## Ethics Statement

Ethical review and approval was not required for the animal study because all data used for this study were collected during clinical treatment and pathological examination and were obtained to diagnose the clinical case. Written informed consent was obtained from the owners for the participation of their animals in this study.

## Author Contributions

MWa planned and supervised the study. SN, AA, MWe, and MWa contributed to conception and design of the study. CP, SK, and PK conducted necropsies. CP helped with interpretation of necropsy reports. SN collected and analyzed the data and wrote the manuscript. All authors contributed to manuscript revision, read, and approved the submitted version.

## Funding

This Open Access publication was funded by the Deutsche Forschungsgemeinschaft (DFG, German Research Foundation) within the programme LE 824/10-1 Open Access Publication Costs and University of Veterinary Medicine Hannover, Foundation.

## Conflict of Interest

The authors declare that the research was conducted in the absence of any commercial or financial relationships that could be construed as a potential conflict of interest.

## Publisher's Note

All claims expressed in this article are solely those of the authors and do not necessarily represent those of their affiliated organizations, or those of the publisher, the editors and the reviewers. Any product that may be evaluated in this article, or claim that may be made by its manufacturer, is not guaranteed or endorsed by the publisher.
