# Supplementary material for: Gastric Ulcers in Alpacas— Clinical, Laboratory, and Pathological Findings
Source: Front Vet Sci. 2022 May 18;9:877257. doi: 10.3389/fvets.2022.877257 (PMC9159277; doi:10.3389/fvets.2022.877257)
Supplement: Supplementary file 1 [file Data_Sheet_1.docx]

Supplementary Material

**S1.** Hematologic reference intervals for alpacas according to Hengrave Burri et al. (1).

| Parameter | Reference intervals | | |
| --- | --- | --- | --- |
|  | Juvenile (<6 months) | Female | Male |
| PCV [l/l] | 0.29-0.37 | 0.26-0.37 | 0.29-0.37 |
| Hemoglobin [g/l] | 134-166 | 110-161 | 127-166 |
| WBC [G/l] | 7.3-16.0 | 8-16 | 9.8-15.8 |
| Lymphocytes [G/l] | 1.4-5.9 | 1.1-5.2 | 1.7-4.5 |
| Segmented neutrophils [G/l] | 2.9-8 | 3.4-9.1 | 4.5-9.3 |
| Band neutrophils [G/l] | 0-0.2 | 0-0.1 | 0-0.1 |
| Eosinophils [G/l] | 0.1-3.1 | 0.8-3.4 | 1-3.6 |
| Basophils [G/l] | 0-0.1 | 0-0.2 | 0-0.3 |
| Monocytes [G/l] | 0.3-0.9 | 0.2-0.9 | 0.1-0.7 |

**S2.** Numerical comparison of average blood values of deceased animals with gastric ulcers and deceased animals without gastric ulcers and discharged animals.

| Parameter | Alpacas deceased with gastric ulcers | | Alpacas deceased  without gastric ulcers | | Alpacas  discharged alive | | Normal alpaca reference interval^a^ |
| --- | --- | --- | --- | --- | --- | --- | --- |
|  | *n* | Mean (SD) | *n* | Mean (SD) | *n* | Mean (SD) |  |
| PCV [l/l] | 36 | 0.25 (0.10) | 105 | 0.26 (0.09) | 165 | 0.26 (0.06) | 0.26-0.37 |
| Hemoglobin [g/l] | 36 | 114.83 (44.62) | 104 | 115.00 (42.83) | 165 | 113.07 (30.00) | 110-166 |
| WBC [G/l] | 35 | 13.64 (15.77) | 98 | 14.87 (10.73) | 159 | 12.20 (4.90) | 7.3-16 |
| Lymphocytes [G/l] | 35 | 1.94 (1.85) | 97 | 2.04 (1.55) | 158 | 2.25 (1.38) | 1.1-5.9 |
| Segmented neutrophils [G/l] | 35 | 7.93 (10.03) | 97 | 10.45 (9.19) | 158 | 8.04 (4.27) | 2.9-9.3 |
| Band neutrophils [G/l] | 35 | 2.67 (5.78) | 97 | 1.45 (1.74) | 158 | 0.81 (0.94) | 0-0.2 |
| Eosinophils [G/l] | 35 | 0.06 (0.10) | 97 | 0.19 (0.46) | 158 | 0.59 (0.83) | 0.1-3.6 |
| Basophils [G/l] | 35 | 0.03 (0.04) | 97 | 0.05 (0.21) | 158 | 0.08 (0.12) | 0-0.3 |
| Monocytes [G/l] | 35 | 0.60 (1.19) | 97 | 0.46 (0.89) | 158 | 0.34 (0.36) | 0.1-0.9 |

*Note.* PCV = Packed cell volume; WBC = White blood count; SD = Standard Deviation

^a^ based on published data (1); combined range of three reference intervals (juvenile, male, female)

**Reference**

**1. Hengrave Burri I, Tschudi P, Martig J, Liesegang A, Meylan M. Neuweltkameliden in der Schweiz. II. Referenzwerte für hämatologische und blutchemische Parameter [South American camelids in Switzerland. II. Reference values for blood parameters]. *Schweiz Arch Tierheilkd* (2005) 147(8):335-43. doi: 10.1024/0036-7281.147.08.335.**
